# Where Is My Food? Brazilian Flower Fly Steals Prey from Carnivorous Sundews in a Newly Discovered Plant-Animal Interaction

**DOI:** 10.1371/journal.pone.0153900

**Published:** 2016-05-04

**Authors:** Andreas Fleischmann, Fernando Rivadavia, Paulo M. Gonella, Celeste Pérez-Bañón, Ximo Mengual, Santos Rojo

**Affiliations:** 1 Botanische Staatssammlung München, Munich, Germany; 2 GeoBio-Center LMU, Center of Geobiology and Biodiversity Research, Ludwig-Maximilians-University, Munich, Germany; 3 Illumina, San Francisco, California, United States of America; 4 Laboratório de Sistemática Vegetal, Instituto de Biociências, Universidade de São Paulo, São Paulo, Brazil; 5 Departamento de Ciencias Naturales y Recursos Naturales / Instituto CIBIO, Universidad de Alicante, Alicante, Spain; 6 Zoologisches Forschungsmuseum Alexander Koenig, Leibniz-Institut für Biodiversität der Tiere, Bonn, Germany; Natural Resources Canada, CANADA

## Abstract

A new interaction between insects and carnivorous plants is reported from Brazil. Larvae of the predatory flower fly *Toxomerus basalis* (Diptera: Syrphidae: Syrphinae) have been found scavenging on the sticky leaves of several carnivorous sundew species (*Drosera*, Droseraceae) in Minas Gerais and São Paulo states, SE Brazil. This syrphid apparently spends its whole larval stage feeding on prey trapped by *Drosera* leaves. The nature of this plant-animal relationship is discussed, as well as the *Drosera* species involved, and locations where *T*. *basalis* was observed. 180 years after the discovery of this flower fly species, its biology now has been revealed. This is (1) the first record of kleptoparasitism in the Syrphidae, (2) a new larval feeding mode for this family, and (3) the first report of a dipteran that shows a kleptoparasitic relationship with a carnivorous plant with adhesive flypaper traps. The first descriptions of the third instar larva and puparium of *T*. *basalis* based on Scanning Electron Microscope analysis are provided.

## Introduction

### Carnivorous plant-animal interactions

Carnivorous plants attract, trap and digest animal prey, benefitting from the end products of digestion in overall growth [1,2]. Carnivory has evolved at least seven times in angiosperms, resulting in multiple trapping strategies: adhesive (“flypaper”) traps, snap-traps, pitfall (“pitcher”) traps, suction traps and eel-traps [3,4]. The last three types are modified hollow tubular leaves with a cavity for animal capture, while the first two represent more exposed, open trap types. Although carnivorous plants occur worldwide, the largest diversity is found in the Southern Hemisphere. Today, approximately 800 species of carnivorous plants are known, almost half of which belong to the adhesive flypaper trap type, including ca. 250 species of sundews (*Drosera*, Droseraceae).

Besides the straight forward predator-prey relationship (and possible pollinator-prey conflicts [5,6]), several commensalistic relationships have been described between carnivorous plants and animals, mainly arthropods. Almost all pitcher plants (i.e. New World Sarraceniaceae and carnivorous Bromeliaceae, and Old World Nepenthaceae and Cephalotaceae) appear to have commensal animal species (infauna) living in the phytotelmata (i.e. fluid-filled aquatic microecosystems) created by their hollow leaves. The pitcher inhabitants range from bacteria to large arthropods such as freshwater crabs, and vertebrates such as frogs, which feed on captured prey or other infauna, depending directly or indirectly on the prey trapped by or merely attracted to these plants [7–19]. Some species are known to live and feed only within the traps of pitcher plants, such as the “*Nepenthes*-crab spiders” of the genus *Misumenops* (Arachnida: Thomisidae), the ant *Camponotus schmitzi* (Hymenoptera: Formicidae), and dipteran larvae of different families with primary saprophagous feeding habits [8,10,11,13,15,16].

Although often ignored in the literature, interactions between animals and carnivorous plants not forming phytotelmata are more frequent than previously thought, especially in carnivorous plants with adhesive traps, which are known to be home to several arthropods that move freely between the sticky tentacles on the leaves. These include capsid bugs (Hemiptera: Miridae), ants (Hymenoptera: Formicidae), mites (Acari: Oribatulidae) and slugs (Gastropoda: Agriolimacidae) which feed as commensals and/or kleptoparasites on prey caught by *Drosera* and *Pinguicula* [20–26]. In a well-studied system from South Africa, the two species of the carnivorous plant genus *Roridula* (Roridulaceae) harbor two mutualistic species of capsid bugs of the genus *Pameridea* (Hemiptera: Miridae), living in a symbiotic relationship with the plant that has been termed “digestive mutualism”: the *Pameridea* bugs feed on prey caught by *Roridula*, while the plant takes up the prey-derived nutrients from the bugs’ feces through its specialized leaf surface [27–30].

### Predatory flower flies

Adults of the family Syrphidae (Insecta: Diptera) are commonly called flower- or hoverflies. They are conspicuous anthophilous Diptera, often mimicking stinging bees or wasps in coloration, appearance and behavior [31,32]. Flower flies are frequently observed on various types of flowers that are often used as mating sites and energy sources, most adults feed on nectar or pollen or both [33–35].

Syrphid larvae are found in various habitats and have diverse feeding habits [36], including species predatory of soft-bodied arthropods, scavengers, saprophages in litter and decaying wood, coprophagous, phytophagous, aquatic detritus feeders, or specialized inquilines in nests of social insects, such as ants, termites, wasps, and bees [37,38].

Approximately 6000 species of flower flies are currently recognized [39,40] and circa one third occur in the Neotropical Region, the richest biogeographic region in terms of taxa and one of the centers of biodiversity of Syrphidae [39,41–43]. The large genus *Toxomerus* is a monophyletic group of predatory flower flies from the subfamily Syrphinae endemic to the New World [39,44], and it is one of the largest and most abundant genera of syrphids in the Neotropics [45,46]. The genus comprises more than 140 known species, mostly from Central and South America, with only 16 species occurring in the Nearctic Region [39,42,47]. In Brazil in particular, 36 species of *Toxomerus* are recorded [48]. Adults have been reported as floral visitors feeding on pollen and nectar of a wide range of plants, including *Drosera* [49,50].

Little is known about the larval biology of *Toxomerus*, and the feeding habits of only about 13 species (less than 10% of the known species) have been described [51–55]. Most *Toxomerus* larvae have been reported to be predators, a feeding mode assumed to be the norm within this genus, as it is in the majority of Syrphinae [56]. However, there are some exceptions in this subfamily, with zoophagous larval habits of some taxa (e.g. a few species of the genus *Allograpta*) have evolved towards phytophagy (leaf miners, stem borers) and/or pollen-feeding [44,57–59]. Larvae of at least three *Toxomerus* species have been discovered to be pollen feeders of several plant families [52,60].

### The discovery of a new carnivorous plant-insect interaction

In 1994, dipteran larvae crawling freely on leaves of *Drosera graomogolensis* were discovered by FR in northern Minas Gerais state, southeastern Brazil, and seen again over the following years at multiple locations and occasions on several *Drosera* species in Minas Gerais and São Paulo states [61] (see Table 1). The larvae were observed to feed on prey captured by the adhesive traps of *Drosera*, but apparently only after the trapped insects were dead. Eventually, the larvae would pupate and their green to brown-black pupae were seen hanging from the lower leaf surfaces of the sundew plants. Larvae and pupae were collected in the field, kept in the laboratory at the University of São Paulo, and the adult flies that emerged were identified as *Toxomerus basalis* (Walker, 1836) [62]. The larval biology of this syrphid species was completely unknown until now, and no dipteran inhabitants of *Drosera* had been reported yet.

**Table 1:** *Drosera* species and respective localities where and when *Toxomerus* larvae and pupae were observed.

Locality (State)	Host Species	Approx. Coordinates	Month/Season
Serra do Cipó (Minas Gerais)	*Drosera chrysolepis* Taub.	19°13'S 43°29'W	
Botumirim (Minas Gerais)	*D*. *graomogolensis* T.Silva; *D*. *spiralis* A.St.-Hil.	16°55'S 43°00'W	September 2011 (dry season)
Grão Mogol (Minas Gerais)	*D*. *graomogolensis*; *D*. *spiralis*	16°35'S 42°54'W	June/September 1994 (dry season); September 2011 (dry season)
Salesópolis (São Paulo)	*D*. *latifolia* (Eichler) Gonella & Rivadavia	23°39'S 45°40'W	September 2011 (dry season)
Conselheiro Pena (Minas Gerais)	*D*. *magnifica* Rivadavia & Gonella	19°19'S 41°35'W	July 2014 (dry season)
Diamantina (Minas Gerais)	*D*. *spiralis*	18°15'S 43°37'W	July 1995 (dry season); February 1997 (wet season)
Milho Verde, Serro (Minas Gerais)	*D*. *spiralis*	18°27'S 43°26'W	May 2007 (wet season/early dry season)
Itacambira (Minas Gerais)	*D*. *grantsaui* Rivadavia; *D*. *spiralis*; *D*. × *fontinalis* Rivadavia	17°04'S 43°19'W	March 1997 (wet season)

## Materials and Methods

### Study material and morphological examination

Pupae of *Toxomerus* found attached to the lower leaf surfaces of *Drosera magnifica* were collected at the Pico do Padre Ângelo (summit of the peak; 1530 m), Minas Gerais state, Brazil, on the 8th of July 2014 (the Instituto Chico Mendes de Conservação da Biodiversidade (ICMBio) issued permits for fieldwork (to PMG), no specific permissions were required to access the locations. The field study did not involve endangered or protected species). Pupae were kept in glass vials with drilled lids, at room temperature, in the dark. Adults emerged within three to six days after collection and were immediately preserved in 96% ethanol. Larvae of different instars—one second instar (L2) larva and three third instar (L3) larvae—were also collected *in situ* from the *Drosera* leaves at the same locality and preserved in 96% ethanol.

Preserved puparia were studied and compared with the larvae and puparia of other *Toxomerus* species, as well as all known preimaginal descriptions in the literature [52,60,63–65]. All larvae were thoroughly examined using a stereomicroscope (LEICA MZ16) and important characters were photographed using a LEICA DFC320 digital camera. Micromorphological studies were made with a Scanning Electron Microscopy (JEOL JSM-5410) equipped with an Oxford CTI500 cryo-preparation assembly as described in Pérez-Bañón *et al*. [66]. Debris adhered to the puparial integument was removed by placing the specimens in an ultrasonic cleaner (P-Selecta Ultrasons 6L) for a few minutes. In the case of pupae, cleaned specimens were examined with SEM but using the less destructive variable-pressure (low vacuum) mode. Larvae dimensions are only an estimate as specimens were not boiled in water previous to fixation in alcohol, a commonly used method explained by Rotheray [36], morphological terminology for larvae and puparia follows Rotheray [36] and Rotheray & Gilbert [64]. The positions of the sensilla are numbered sequentially from the dorsal to the ventral surface for each segment [36].

The adult specimens that emerged from the field-collected pupae in Brazil are deposited in the collection of the Zoologisches Forschungsmuseum Alexander Koenig (Bonn, Germany; ZFMK) (numbers ZFMK-DIP-00012164, -00012165, -00012166, -00012167). Studied larvae are deposited as follows: one L3 larva and one L2 larva in the ZFMK collection (ZFMK-DIP-00012168, -00012169), and one L3 larva and three puparia used for the micromorphological studies in the Entomological collection of the University of Alicante (Alicante, Spain; CEUA).

### Molecular protocols

Whole specimens (adults and larvae) were used for DNA extraction. Extractions were carried out using the NucleoSpin Tissue DNA Extraction kit (Machery-Nagel, Düren, Germany) following the manufacturer's instructions; samples were resuspended in 100 μl ultra-pure water. Entire specimens were preserved and labeled as DNA voucher specimens for the purpose of morphological studies and deposited at the Zoological Museum Alexander Koenig (ZFMK), with the unique identifiers ZFMK-DIP-00012164 to ZFMK-DIP-000169.

DNA primers and PCR amplification protocols for mitochondrial COI were the same as described in [56, 67]. Amplified DNA was electrophoresed on 1.5% agarose gels for visual inspection of amplified products. PCR products were enzymatically treated with ExoSap-IT (USB, Cleveland, OH, USA) and then sequenced in both directions, using the PCR primers. The sequences were edited for base-calling errors and assembled using Geneious version 7.1.3 (Biomatters Ltd.). All sequences were submitted to NCBI GenBank (accession numbers KU216212 to KU216216).

## Results

### Species involved

**Fig 1:**
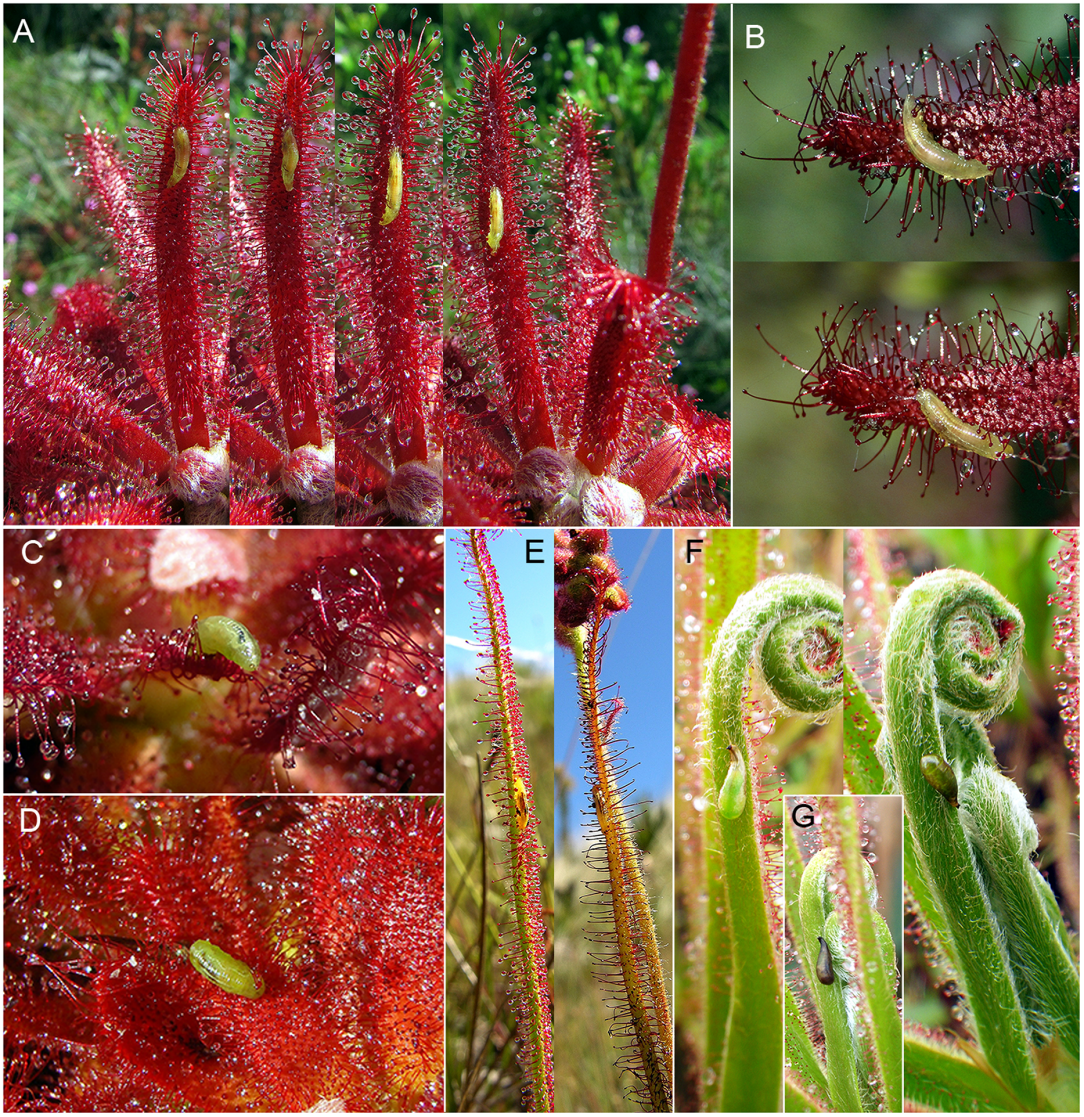
*Toxomerus basalis* larvae and puparia *in situ*. A, B. Larva moving on the glandular leaves of *Drosera graomogolensis*, Grão Mogol, Minas Gerais (time lapse between the single shots of the photo sequence A less than 1 minute). C, D. Larvae on the glandular lamina of *Drosera graomogolensis*, Botumirim, Minas Gerais (note the larval posterior spiracles in C). E. Larvae on *Drosera spiralis*, Milho Verde, Minas Gerais. F, G. Puparia on *Drosera magnifica*, Pico do Padre Ângelo, Minas Gerais. A by Paulo Gonella, B, C by Adilson Peres, D by Nílber Silva, E, F by Fernando Rivadavia, G by Carlos Rohrbacher.

Larvae and pupae were observed on six different *Drosera* species at several locations (Fig 1; Table 1). Three females and one male emerged from the pupae collected at the Pico do Padre Ângelo, and all adults were identified as *Toxomerus basalis* using different identification keys [47, 48]. DNA barcodes were obtained for four adults and one L3 larva, each of 696 nucleotides long. Two different haplotypes were found among the obtained DNA barcodes, with an uncorrected pairwise distance of 1.58%. Specimens ZFMK-DIP-00012165 (GenBank accession KU216213) and ZFMK-DIP-00012166 (KU216214) share the same haplotype, while the COI sequence of the L3 larva (ZFMK-DIP-00012169; KU216216) is identical to the sequences of the other two adults, ZFMK-DIP-00012164 (KU216212) and ZFMK-DIP-00012167 (KU216215). This corroborates that larvae and pupae taken from the *Drosera* plants belong to the same species, *T*. *basalis*.

*Toxomerus basalis* was originally described from São Paulo, and adults have been additionally collected from Rio de Janeiro, São Paulo and Santa Catarina states [48]. Thus, records from Pico do Padre Ângelo represent the first record of *T*. *basalis* for Minas Gerais state.

### Description of immature stages of *Toxomerus basalis*

#### Third larval instar

**Fig 2:**
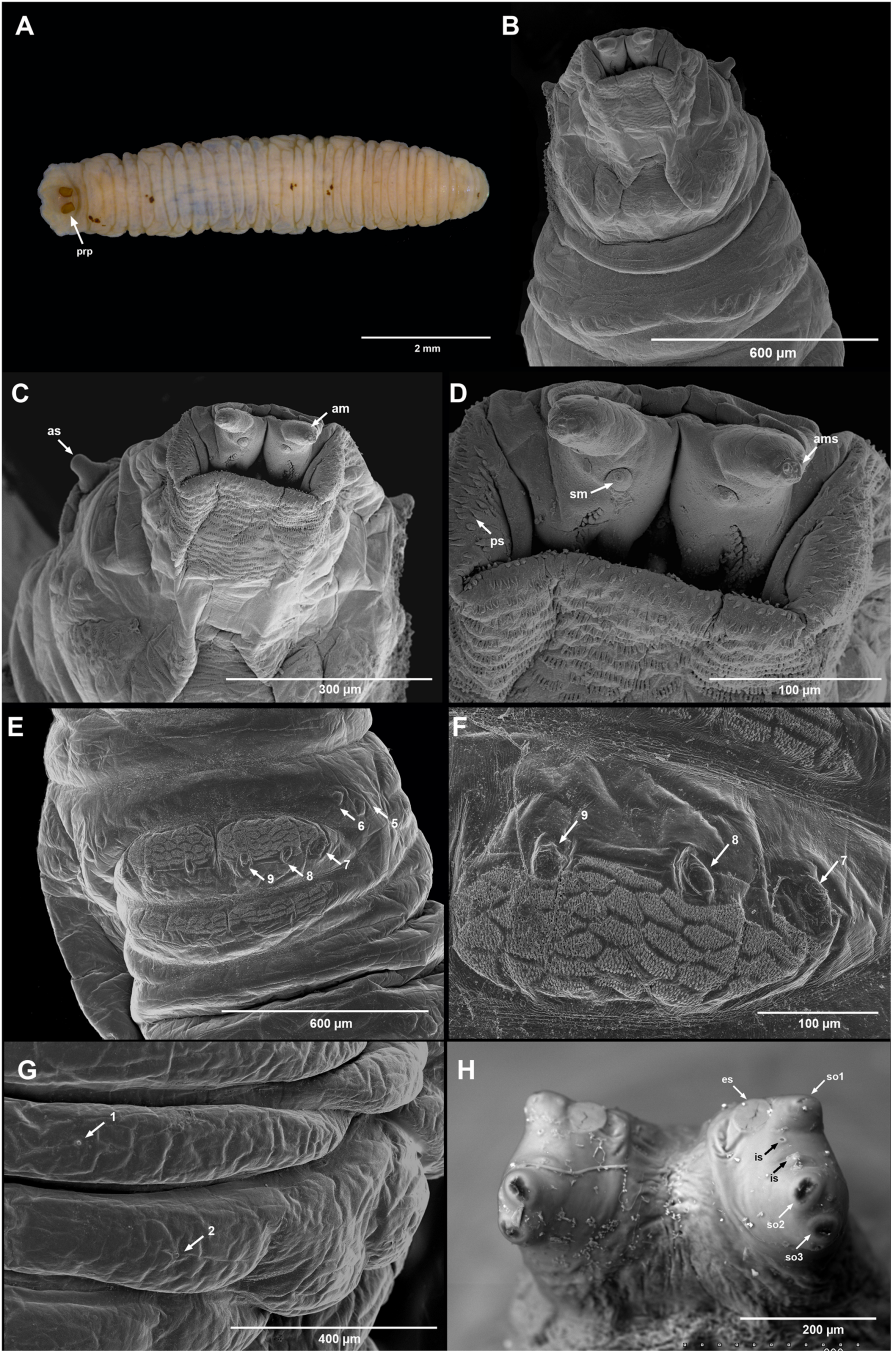
Larva of *Toxomerus basalis*. A. dorsal view. B–D. head and prothorax. E. fourth abdominal segment, ventral view. F. fourth pair of prolegs, with ornamentation. G. fifth abdominal segments with the 1st pair of segmental sensilla on second fold and 2nd pair of segmental sensilla on third fold, dorsal view. H. posterior breathing tube; *prp*: posterior breathing tube; *as*: anterior respiratory process; *am*: antenno-maxillary organs; *ams*: sensilla on top of the antenno-maxillae organs; *ps*: prothorax spicules; *sm*: pair of sensilla located above the mouth and below the antenno-maxillary organs; *es*: ecdysial scars; *so1*: spiracular opening I; *so2*: spiracular opening II; *so3*: spiracular opening III; *is*: interspiracular setae; numbers showing the position of segmental sensilla.

Length 8–9 mm, maximum width 1.0–2.0 mm. Oval in cross-section with a flattened ventral surface, tapering anteriorly and slightly truncate posteriorly (Fig 2A). The larvae are usually light green in color, but often yellowish or even orange, with black and/or reddish or yellow lateral stripes along their length (Fig 1). Dorsal habitus wrinkled, all segmental sensilla are much reduced, papilliform and without segmental spines (Fig 2A, 2E and 2G). Dorsal surface smooth, without integumental vestiture (except the dorsal surface of prothorax). Posterior breathing tube short, spiracular plates on a slightly projecting fleshy bar and not joined by sclerotization (Fig 2A). Head much reduced (Fig 2B and 2C), mouthparts adapted for piercing-feeding [68] with distinctive features of predacious syrphid larvae. Lateral margins of mouth present a pair of triangular pointed sclerites only slightly sclerotized. Mandibles slender, extended anteriorly into the fleshy projections on which antenno-maxillary organs are situated. Head skeleton with labrum and labium strongly sclerotized and sharply pointed, which are curved respectively dorsal and ventral. Antenno-maxillary organs well developed (Fig 2C and 2D), located on fleshy projections and separated by a groove between them acting as a guide for the retractile apex of the head skeleton. Seven pairs of locomotory prominences present on abdominal segments 1–7. Prolegs with a band of small backwardly directed spicules just before the sensilla (11–9), aggregated in a polygonal pattern (Fig 2E and 2F). A second band of small backwardly directed spicules aggregated in a polygonal pattern on fold just behind the prolegs (Fig 2F).

#### Puparium

Length 5–6 mm, maximum width 1.50–2.0 mm. Pear-like, sub-cylindrical in cross-section. Anterior extreme truncated, slightly tapering posteriorly and flattened ventrally. Dark green coloration, similar to pupae of *Toxomerus floralis* [60]. Integumental vestiture and segmental spines absent. Color of empty puparium light brown. Posterior breathing tube completely sclerotized, including the projecting fleshy bar that joins the spiracular plates (Fig 2H).

Although a large number of *Toxomerus* species have been described, biology and feeding habits of only 13 species are known, and only five of them have some data on preimaginal morphology [52,60,63], making *Toxomerus* one of the poorest known genera of Syrphinae [39]. Most of these descriptions lack diagnostic characters or are very general, and chaetotaxy studies of *Toxomerus* are only present in a couple of publications [52,64]. Based on these previous works and the present study, a diagnostic characteristic for *Toxomerus* larvae may be the disposition of the spiracular plates, which are on a slightly projecting fleshy bar and are not joined by sclerotization (Fig 2A), as well as the pattern of the spiracular slits, with slits II and III clearly separated from slit I (Fig 2F) (see Fluke [65] for an explanation).

The larva of *T*. *basalis* is similar to other known larvae of *Toxomerus* species, but can be recognized by the ornamentation of the ventral abdominal segments with two band of small backwardly directed spicules aggregated in a polygonal pattern, one band just before the sensilla (11–9) on the prolegs and a second band just behind the prolegs (Fig 2E and 2F). Another diagnostic character is the almost total absence of ornamentation on the dorsal surface, with the segmental sensilla reduced to very small papillae and without setae (Fig 2G). The reduction of ornamentation might be related with its mode of living, as a smooth body surface could facilitate the movement on the glandular adhesive leaves of *Drosera*. The well-studied larvae of *T*. *politus* and other species have segmental sensilla with setae on the dorsal surface.

The pointed and heavily sclerotized ends of the labrum and labium of *T*. *basalis* resemble those of entomophagous syrphinae larvae. The labrum and labium protrudes along the groove of the dorsal lip and pierces the prey. Nevertheless, the cephalopharyngeal skeleton of *T*. *basalis* shows some morphological features that are atypical among Syrphinae predatory larvae. These features are the dorsally curved apex of the labrum and ventrally curved apex of the labium. These characteristics are shared with phytophagous species such as *Fazia micrura* and *Toxomerus apegiensis*. According to some authors [52,59] these features may be adaptations to a pollen- feeding mode of the larvae, and the outwardly curved labrum and labium assist in breaking into the flowers of the host plant to access pollen. These features have also been observed in *T*. *floralis* [60] but are curiously absent in other species, e.g. *T*. *politus*.

### Larval behavior and life cycle

Larvae of different sizes were observed on the lower and upper surfaces of the *Drosera* leaves (however usually not more than a single larva on a small percentage of plants per population), where they crawled freely and did not appear to adhere to the viscous water-based mucilage secreted by the glandular emergences (Fig 1A–1E). Apparently, this species spends its entire larval life on the leaves of the sundew. Most often, the larvae were seen moving, resting, or feeding among the glandular emergences of the sundew leaves, where they are well-camouflaged on *Drosera* species with yellow-green leaves (Fig 1E), and where they fed on the immobilized (or dead) prey captured by the sundews. The return of nutrients to the plant from larval excretions is not likely, as Syrphinae larvae do not defecate during their feeding period until they pupate (which, in *T*. *basalis*, happens on the lower leaf surface that is free of digestive glands). Instead, they accumulate in their hind gut the dark-colored remnants of prey digestion, where these even contribute to the larva’s cryptic color pattern [38]. Therefore, the larvae live on the plant as so-called kleptoparasites (following the definition of Hamilton [69]), as they abstract part of the arthropod prey that was caught by the plant for its own nutrient supply. When disturbed, the larvae crawled on the lower surface of the leaves, or towards the base of the leaves, into the center of the sundew rosettes. Pupae were found attached to the lower (non-glandular) leaf surfaces of *D*. *magnifica* and *D*. *spiralis*.

Dynamic fluctuations of larval abundance were observed at some sites, where *Toxomerus* larvae were sometimes not found in sundew populations where they had been noticed in previous years. This suggests that there may be certain times of the year when larvae are predominately present, although no pattern has yet been identified. Generally, larvae were observed on *Drosera* leaves both during the dry and wet season (Table 1). However, this may vary according to geography, host sundew species, available insect prey and possibly even *Toxomerus* species (in case further species other than *T*. *basalis* are found to share this feeding strategy).

## Discussion

Kleptoparasitism, i.e. to feed on prey or food prepared or caught by other animals (or carnivorous plants), is well known in Diptera. Several dipteran families include taxa that have larvae and/or adults showing kleptoparasitic behavior with different feeding strategies [70–72]. *Toxomerus* is also not the first reported syrphid having larvae as inhabitants of carnivorous plants in general. Larvae of the Old World genus *Nepenthosyrphus* (Syrphidae: Eristalinae) develop inside the pitcher trap fluid of *Nepenthes* in South-East Asia, where they are aquatic sit-and-wait predators of other pitcher infauna [13,73,74].

Several examples of kleptoparasitic and commensalistic behavior are known where predators feed on immobilized insects captured by sticky plant surfaces, including on carnivorous plants. Most well-known examples involving insects are capsid bugs (Hemiptera: Miridae), ranging from opportunistic feeders to mutualistic symbionts [4,27,75,76]. The abundance of dead and decaying animals turns such traps into attractive habitats for different organisms. Zamora & Gomez [25] argued that “carnivorous plants provide large quantities of high quality food items to dietary opportunistic animals”, and Zamora [24] concluded that kleptoparasitic interactions are favored by the prolonged time which prey remains on (or inside) the leaves of carnivorous plants. This is especially true for carnivorous plants with adhesive traps, such as *Drosera*, where prey is presented more or less freely exposed on the leaf surfaces, although the leaf blades of some species are able to fold over their prey to reduce loss from rain, commensals and kleptoparasites [1,4]. Interestingly, the larvae of *Toxomerus* were observed most frequently on species with erect, less mobile laminae (e.g. *D*. *spiralis*) but also on certain species with very mobile leaves (e.g. *D*. *latifolia*). With the exception of the small-sized *D*. *grantsaui* and *D*. × *fontinalis* (which were growing very close to large *D*. *spiralis*), all other taxa on which the larvae were observed are comparatively large and robust. Larger sundew species may generally capture more and larger prey, offering a more stable and safe environment for the larva, but it is also possible that, contrary to rosetted *Drosera* species with leaves flat on the ground, those with semi-erect leaves may either offer more exposed oviposition sites for the adult flies, or better protection from ground-dwelling predators.

Although oviposition of *T*. *basalis* females has not yet been observed, it is most likely that the adults land on the petioles, lower leaf surfaces, young leaves in bud, or on the flower scapes when depositing their eggs directly on the plant, in order to avoid becoming trapped by the glandular tentacles of the lamina. Similarly, the herbivorous caterpillars of the sundew plume moth (*Buckleria*) hatch from eggs deposited by the adult moths on the non-glandular parts of its host plant *Drosera*, namely flower scapes, seed capsules or petioles [77]. Due to the low dispersal capacity of the syrphid larvae, the choice of oviposition location is crucial for predatory syrphines, because the quality of oviposition sites can greatly affect the progeny growth and the survival of the offspring [78]. A targeted oviposition on the host plant of the preferred prey is known from many aphidophagous syrphids, but several factors may affect the choice of the oviposition site [79].

Predatory syrphid larvae are often well camouflaged by their coloration, which serves to break up the body outline on the surfaces they live upon (“crypsis” sensu Rotheray [31]). This is also the case with the *Toxomerus* larvae observed on *Drosera* leaves, which are well camouflaged at least on those sundew species with greenish leaves (Fig 1E).

The viscoelastic, aqueous polysaccharide mucilage secreted by *Drosera* glands [1,80,81] has only a limited retention capacity, which delimitates possible prey size for the plant as it allows larger, more vigorous insects to escape from the sticky traps [82,83]. This limited retention capacity is probably also what enables the apodal *Toxomerus* larvae to freely move on the adhesive sundew leaves. Further, syrphid larvae secrete a watery fluid that lubricates their ventral body surface for movement [38]—this fluid secretion might also prevent the larvae from adhering to the *Drosera* glands. Other dipteran larvae are also well-known to be able to move on rather sticky surfaces, such as the gastropod-preying larvae of “snail-killing marsh flies” (Sciomyzidae; [84]), most necrophagous flies [85], and the larvae of syrphids living on viscous surfaces such as resinous exudates of trees [86]. For locomotion on sticky surfaces, the strength of viscoelastic glue also depends on animal dynamics [87], hence the ability to move through sundew mucilage could also rely on a special locomotion behavior of the larvae. Little is known about locomotion of *Toxomerus* larvae in general in order to have enough data for comparison, and the larvae of *T*. *basalis* did not show any avoidance strategies to overcome the adhesive sundew tentacles. The *Toxomerus* larvae described here are also apparently not adversely affected by the numerous digestive enzymes present in the mucilage of *Drosera*. This is not surprising due to the general design and cuticle of acephalous dipteran larvae—a fact that is well-known from various other dipteran larvae (including syrphids) which live in hostile, digestive fluids such as acidic vertebrate stomach fluid or pitcher plant digestive fluids [13,88,89]. Adlassnig *et al*. [90] state incorrectly that “no animals are known to use the traps of *Drosera* as a permanent habitat”, and they argued that this was most likely due to naphthoquinones like droserone, which are secreted with the mucilage and act as insect repellants. However, not only do commensals and kleptoparasites live on *Drosera*, as summarized here, but also arthropod herbivores such as caterpillars [77,91], and phytoparasites such as aphids [92,93] are frequently encountered feeding on *Drosera*, despite the phytochemical defenses.

Usually, predatory Syrphinae larvae feed on relatively immobile or slow moving, soft-bodied prey such as aphids or immature stages of other arthropods [36,51]. Thus, it is not unexpected that immobilized arthropods stuck to adhesive plant surfaces would prove to be an easily exploitable food source. The adaptation to new food sources might have become necessary in tropical latitudes, as aphids, the predominantly preferred prey of predatory syrphid larvae, are largely absent in the Neotropics [94]. This might have driven predatory Neotropical syrphids to evolve different, alternative feeding strategies exploiting an unusually wide range of prey [95,96] and other available food sources such as pollen-feeding or phytophagy [52,59].

Larvae of *Toxomerus basalis* likely feed on any available insect prey captured by the sundew leaves, although the majority of the *Drosera* prey spectrum identified thus far from the few Brazilian species studied consists mostly of small to medium sized adult midges, mosquitos and gnats [61,97]. It is therefore likely that the larval diet of *T*. *basalis* consists largely of “nematoceran” adult flies. Although the larval biology of only a handful of *Toxomerus* species is known, the diversity of prey taxa is extraordinary [51,53–55,98]. The larvae of at least one species of predatory flower fly feed on adult diptera [96], and larvae of a few species (including *Toxomerus geminatus*) prey on larvae of small dipterans, butterflies or beetles [51,98]. However, all known predatory syrphine larvae studied thus far feed on living prey. Here, the first Syrphinae larva feeding on immobilized or dead prey is reported. With this new trophic relationship as a kleptoparasite on a sticky carnivorous plant, Neotropical syrphines become the dipteran family with the most diverse larval feeding strategies, including pollen-feeding, phytophagy, predation on a wide range of prey, and kleptoprasitism/saprophagy. The evolutionary scenario for the genus *Toxomerus* is even more fascinating after Jordaens *et al*. [60], who showed a recent switch of feeding-mode in an African neozoan *Toxomerus* species, whose larvae are pollenivorous in the Ethiopian region, but which have a predaceous habit in its Neotropical region of origin.

## Conclusions

It is possible that *Toxomerus* species with larvae living on *Drosera* (and other viscous plants) are more common and widespread than what we have observed. It is also probable that the larvae may occur on more sundew species in Brazil and South America, and that more than one species of *Toxomerus* is involved. Similar syrphid larvae have casually been observed on two different species of the glandular-adhesive, non-carnivorous genus *Chamaecrista* (Fabaceae: Caesalpinioidae) in Minas Gerais state. We are confident that *T*. *basalis* may have a broader distribution in Southeastern Brazil, as shown by our records of larvae on *Drosera* at seven different localities, which lie widely scattered in central Minas Gerais and São Paulo states. We must point out that some of the larvae observed in the field might belong to different species of *Toxomerus* (or even to other Syrphidae genera), but at least the record from Pico do Padre Ângelo, from where the hatched adult specimens originate, represents the first record of *T*. *basalis* for Minas Gerais.

This is the first record of dipteran larvae living as kleptoparasites of a sticky carnivorous plant, and it is the first report of a syrphid species using this unique feeding strategy. Thus far, the knowledge of Neotropical carnivorous plant infauna was limited to pitcher plants (the sarraceniacean genus *Heliamphora* and the carnivorous bromeliads *Brocchinia* and *Catopsis* [9,99,100]).
